# The Clinicopathological Significance of Tumor Cell Subtyping in Appendiceal Neuroendocrine Tumors: A Series of 135 Tumors

**DOI:** 10.1007/s12022-024-09813-4

**Published:** 2024-06-04

**Authors:** Ozgur Mete, David W. Dodington, Daniel L. Shen, Sylvia L. Asa

**Affiliations:** 1 Department of Pathology, University Health Network, Toronto General Hospital, 200 Elizabeth Street, 11th floor, Toronto, ON Canada M5G 2C4; 2 Department of Laboratory Medicine and Pathobiology, University of Toronto, Toronto, ON Canada; 3 Endocrine Oncology Site, Princess Margaret Cancer Centre, Toronto, ON Canada; 4 Department of Pathology, Institute of Pathology, University Hospitals Cleveland, Case Western Reserve University, 11100 Euclid Avenue, Room 204, Cleveland, OH 44106 USA; 5 Department of Pathology, University of Texas Southwestern, Dallas, TX USA

**Keywords:** L-cell, EC-cell, Serotonin, Cell subtyping, SATB2, Appendix, Neuroendocrine tumor, Appendiceal neuroendocrine tumor

## Abstract

Appendiceal neuroendocrine tumors (NETs) are common and often are identified as incidental lesions at the time of appendectomy. The guidelines for management are based on tumor size, degree of invasion, and the Ki67 proliferation index. Most small bowel NETs are composed of serotonin-producing EC-cells, but there are multiple other neuroendocrine cell types. In the rectum, there are L-cell tumors that express peptide YY (PYY), glucagon-like peptides (GLPs), and pancreatic polypeptide (PP); they are thought to have a better prognosis than serotonin-producing tumors. We investigated whether the appendix has distinct neuroendocrine tumor types based on cell type and whether that distinction has clinical significance. We collected 135 appendiceal NETs from the pathology archives of UHN Toronto and UHCMC (Cleveland). We analyzed the expression of biomarkers including CDX2, SATB2, PSAP, serotonin, glucagon (that detects GLPs), PYY, and pancreatic polypeptide (PP) and correlated the results with clinicopathologic parameters. Immunohistochemistry identified three types of appendiceal NETs. There were 75 (56%) classified as EC-cell tumors and 37 (27%) classified as L-cell tumors; the remaining 23 (17%) expressed serotonin and one of the L-cell biomarkers and were classified as mixed. EC-cell tumors were significantly larger with more extensive invasion involving the muscularis propria, subserosa, and mesoappendix compared with L-cell tumors. Mixed tumors were intermediate in all of these parameters. Both EC-cell and mixed tumors had lymphatic and/or vascular invasion while L-cell tumors had none. Unlike EC-cell NETs, L-cell tumors were not associated with lymph node metastasis. Tumor type correlated with pT stage and the only patient with distant metastatic disease in this series had an EC-cell tumor. Our study confirms that appendiceal NETs are not a homogeneous tumor population. There are at least three types of appendiceal NET, including EC-cell, L-cell, and mixed tumors. This information is important for surveillance of patients, as monitoring urinary 5HIAA levels is only appropriate for patients with serotonin-producing tumors, whereas measurement of GLPs and/or PP is more appropriate for patients with L-cell tumors. Our data also show that tumor type is of significance with EC-cell tumors exhibiting the most aggressive behavior.

## Introduction

Appendiceal neuroendocrine tumors (NETs) are common well-differentiated epithelial neuroendocrine neoplasms that often are identified as incidental lesions at the time of appendectomy. Several guidelines for management of appendiceal NETs have been developed for risk stratification and/or to determine the need for extended surgery such as hemicolectomy [1–9]. While there are still open questions in the management of select patients, most guidelines discuss the impact of age (pediatric *vs* adult), tumor location within the appendix, tumor size, degree of invasion, lymph node metastasis, and the WHO tumor grade including the Ki67 proliferation index [6]. However, there has not been a role for tumor subtype classification in assessing the risk stratification and determining management for patients with these neoplasms. Data on hormone immunohistochemistry are scant with a recent study showing lack of nodal metastases in patients with glucagon-positive appendiceal NETs compared to serotonin-positive tumors [10].

In other body sites, neuroendocrine tumors have been the focus of pathologists for subtyping based on cell of origin and hormone production. The most elaborate example is the pituitary where tumor cytogenesis and degree of cell type differentiation have proven to be more valuable than tumor grade [11]. At the other end of the spectrum, the small bowel is the site of an almost homogenous tumor type, since the vast majority of small bowel NETs are composed of serotonin-producing EC-cells. In the rectum, it is now recognized that there are serotonin-producing EC-cell NETs as well as L-cell NETs that express glucagon-like peptides (GLPs), peptide YY (PYY), and pancreatic polypeptide (PP) [12–14]; the distinction has been shown to be clinically important since L-cell NETs tend to have a better prognosis than serotonin-producing EC-cell tumors [12–14].

We investigated whether the appendix has distinct neuroendocrine tumor subtypes based on cell type and assessed the clinicopathological correlates of cell type and hormone production in a series of 135 well-characterized appendiceal NETs.

## Materials and Method

### Cohort and Clinicopathologic Characteristics

With institutional research ethics approval, a retrospective review of the pathology files was performed to identify well-differentiated neuroendocrine tumors of the appendix diagnosed between 2004 and 2020 at the University Health Network (UHN) Toronto (*n* = 72) and the University Hospitals Cleveland Medical Center (UHCMC) (*n* = 63). Pathologic variables including Ki67 labeling index, tumor size, extent of invasion, presence of lymphatic or vascular invasion, presence of perineural invasion, and margin status were obtained from the diagnostic reports. Tumors were staged according to the AJCC Cancer Staging Manual 8th edition. Clinical, radiological, and biochemical recurrence data were obtained from the clinical chart.

### Immunohistochemistry and Tumor Classification

**Fig. 1:**
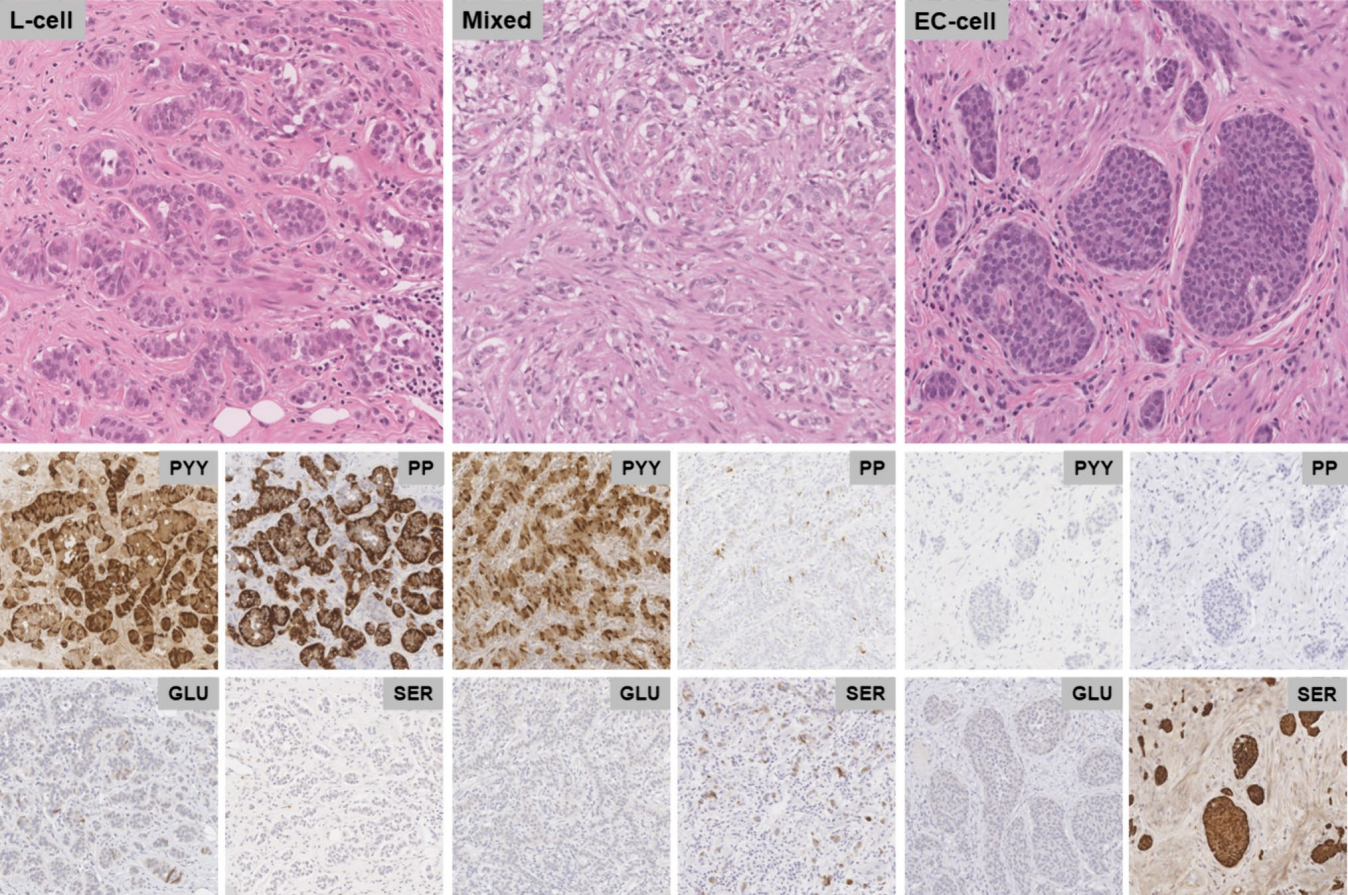
Immunohistochemical characterization of appendiceal neuroendocrine tumors. Representative H&E micrograph of L-cell (left panel), mixed (middle panel), and EC-cell (right) appendiceal neuroendocrine tumors with accompanying immunohistochemical staining results for peptide YY (PYY), pancreatic polypeptide (PP), glucagon (GLU), and serotonin (SER)

The slides generated at the time of diagnostic workup were reviewed including H&E-stained slides, immunohistochemical stains for chromogranin, Ki67, markers of L-cell differentiation (PYY, pancreatic polypeptide (PP), and glucagon (GLU)), markers of EC-cell differentiation (serotonin (SER)), and cell differentiation markers (caudal type homeobox 2 (CDX2), prostatic acid phosphatase (PSAP), and special AT-rich sequence-binding protein 2 (SATB2)). The routine workup of tumors at each institution was similar except for PSAP, which was routinely performed at the UHN, and SATB2, which was routinely performed at UHCMC. The technical details of immunohistochemical staining techniques varied by institution and over time. For cases with an available paraffin block, stains for PYY, PP, GLU, SER, CDX2, PSAP, and SATB2, which were not performed at the time of the initial diagnostic workup, were completed as part of the study (*n* = 29 for UHN, *n* = 46 for UHCMC). Staining in tumor cells was recorded in a binary fashion as either positive or negative. Tumors that stained positive for L-cell markers and negative for EC-cell markers were classified as L-cell tumors, while those staining positive EC-cell markers and negative for L-cell markers were classified as EC-cell tumors. Tumors that were stained for any combination of L-cell and EC-cell markers, regardless of the proportion, were classified as mixed tumors (Fig. 1).

### Statistical Analysis

The clinical and pathological characteristics of L-cell, EC-cell, and mixed tumors were compared using the chi-square test or Fischer’s exact test when the expected counts were less than five. Analysis was performed using SPSS version 26. Statistical significance was defined as *p* < 0.05.

## Results

A total of 135 well-differentiated NETs of the appendix were identified in 110 appendectomy specimens and 25 bowel resections. Among these, 37 (27%) were classified as L-cell, 75 (56%) were classified as EC-cell, and 23 (17%) were classified as mixed. The proportion of cases staining positive for each immunohistochemical marker is shown in Table 1. Almost all tumors stained positive for CDX2 and SATB2, while PSAP was positive in 82% of cases examined. Most L-cell tumors expressed PYY (91%) and GLU (89%), and a smaller number also expressed PP (36%). For mixed tumors, the most commonly expressed L-cell marker was PYY (82%), followed by PP (52%) and GLU (48%). By definition, all mixed and EC-cell tumors expressed SER.

**Table 1:** Immunohistochemical profile of appendiceal neuroendocrine tumors

	**L-cell (*n* = 37)**	**Mixed (*n* = 23)**	**EC-cell (*n* = 75)**
Cell development biomarkers			
CDX2	35/36 (97%)	23/23 (100%)	72/72 (100%)
SATB2	17/17 (100%)	7/7 (100%)	38/38 (100%)
PSAP	11/17 (65%)	15/17 (88%)	25/28 (89%)
Markers of L-cell differentiation			
PYY	31/34 (91%)	18/22 (82%)	0/71 (0%)
PP	13/36 (36%)	12/23 (52%)	0/72 (0%)
Glucagon	32/36 (89%)	11/23 (48%)	0/68 (0%)
Markers of EC-cell differentiation			
Serotonin	0/37 (0%)	23/23 (100%)	75/75 (100%)

Data represent counts (proportions)

The clinical and pathologic characteristics by tumor type are shown in Table 2. While there was no association between age and tumor type, there was a higher proportion of females with EC-cell tumors compared to L-cell and mixed types. EC-cell tumors were significantly larger than L-cell tumors, and mixed tumors were intermediate in size between the two. Overall, both EC-cell and mixed tumors demonstrated more extensive invasion compared to L-cell tumors. A greater proportion of EC-cell and mixed tumors extended into the muscularis propria compared to L-cell tumors, while a greater proportion of EC-cell tumors involved the subserosa or mesoappendix. A greater proportion of mixed tumors extended to serosa or adjacent tissue compared to L-cell tumors. EC-cell tumors also tended to extend more frequently to serosa or adjacent tissue; however, this did not reach statistical significance. Lymphatic and/or vascular invasion were more common in both EC-cell and mixed tumors compared to L-cell tumors, while perineural invasion was more common only in mixed tumors.

**Table 2:** Clinicopathological characteristics and outcomes of the study cohort

	**L-cell (*n* = 37)**	**Mixed (*n* = 23)**	**EC-cell (*n* = 75)**	***p* value**
Age				0.27
< 25 years	12 (32%)	5 (22%)	14 (19%)	
25–49 years	16 (43%)	7 (30%)	32 (43%)	
≥ 50 years	9 (24%)	11 (48%)	29 (39%)	
Sex				0.003
Female	18 (49%)^a^	13 (57%)^a^	59 (79%)^b^	
Tumor grade				0.86
Grade 1	33 (89%)	19 (83%)	63 (84%)	
Grade 2	4 (11%)	4 (17%)	11 (15%)	
Grade 3	0 (0%)	0 (0%)	1 (1%)	
Tumor size				0.000001
< 0.5 cm	27 (73%)^a^	11 (48%)^b^	17 (23%)^c^	
0.5–0.9 cm	9 (24%)	4 (17%)	28 (37%)	
1.0–2.0 cm	1 (3%)^a^	8 (35%)^b^	22 (29%)^b^	
> 2.0 cm	0 (0%)^a^	0 (0%)^a,b^	8 (11%)^b^	
Tumor extent				0.00000002
Lamina propria/submucosa	13 (35%)^a^	9 (39%)^a^	4 (5%)^b^	
Muscularis propria	17 (46%)^a^	4 (17%)^b^	17 (23%)^b^	
Subserosa or mesoappendix	6 (16%)^a^	5 (22%)^a^	47 (61%)^b^	
Serosa or adjacent tissue	1 (3%)^a^	5 (22%)^b^	8 (11%)^ab^	
Margin status				0.45
Positive margins	0 (0%)	1 (4%)	4 (5%)	
Lymphatic or vascular invasion				0.00009
Present	0 (0%)^a^	7 (30%)^b^	22 (29%)^b^	
Perineural invasion				0.010
Present	2 (5%)^a^	8 (35%)^b^	11 (15%)^a^	
pT category				0.0000006
pT1	31 (84%)^a^	12 (52%)^b^	24 (32%)^b^	
pT2	0 (0%)	0 (0%)	0 (0%)	
pT3	6 (16%)^a^	6 (26%)^a^	43 (57%)^b^	
pT4	0 (0%)^a^	5 (22%)^b^	8 (11%)^b^	
pN category				0.034
pN1	0 (0%)^a^	4 (40%)^b^	6 (23%)^ab^	
pM category				N/A
pM1	0 (0%)	0 (0%)	1 (1%)	
Tumor recurrence				0.76
Yes	0 (0%)	1 (5%)	2 (3%)	

Data represent counts (proportions). Values with different superscript letters are significantly different within a row (*p* < 0.05)

In terms of tumor staging, EC-cell and mixed tumors were of significantly higher pathologic stage compared to L-cell tumors. Specifically, a greater proportion of pT3 tumors were EC-cell tumors and a greater proportion of pT4 tumors were either EC-cell or mixed tumors. Pathological evaluation of lymph nodes was performed in 45 cases (*n* = 9 for L-cell, *n* = 10 for mixed, *n* = 26 for EC-cell). Nodal status was obtained in 13 of these cases from a subsequent right hemicolectomy performed for staging and/or treatment purposes. Lymph node metastasis (pN1) was found in mixed tumors and EC-cell tumors but none was found in patients with L-cell tumors. A single patient with an EC-cell tumor had pathologically confirmed metastasis to the liver at the time of staging.

Clinical follow-up data were available for 105 cases (*n* = 24 (65%) for L-cell, *n* = 61 (81%) for EC-cell, *n* = 20 (87%) for mixed). The median follow-up time was 27 months and ranged from less than 1 month to 141 months. Tumor recurrences were identified in two cases of EC-cell tumors (one biochemical and one with liver metastasis) and in one case of mixed tumor (biochemical recurrence). No recurrences were seen in L-cell tumors.

## Discussion

The role of tumor cell subtype in classification, assessing the risk stratification, and determining the management of appendiceal NETs has not been well defined. In this study, we show that immunohistochemical staining can be used to subclassify appendiceal NETs into at least three subtypes that have distinct clinical and prognostic features. Just over half of these tumors were EC-cell tumors that express serotonin; they resemble EC-cell tumors of the small bowel. Around 30% of appendiceal NETs were L-cell tumors that have a tubular architecture and correspond to the tumor previously known as “tubular carcinoid” [15]. These tumors may be negative for chromogranin [16], and therefore, they may be misdiagnosed in the absence of other stains. Like L-cell tumors elsewhere in the gastrointestinal tract, they can express PP, PYY, and/or glucagon-like peptides that are detected by antibodies to glucagon. The analysis showed that PYY served as the best biomarker of these tumors, being expressed in 91% of L-cell appendiceal NETs. Interestingly, we also detected a significant proportion of tumors (17%) that show evidence of mixed L- and EC-cell differentiation based on overlapping immunoprofiles with expression of serotonin as well as one or more of PP, PYY, and glucagon. All of these subtypes of appendiceal NETs express CDX2 and SATB2, consistent with origin in the distal gastrointestinal tract [17], and PSAP expression was also frequent.

The analysis of clinicopathological features showed interesting correlations between cell type and behavior. EC-cell tumors were significantly larger with more extensive invasion and more often involved the muscularis propria, subserosa, and mesoappendix compared with L-cell tumors. The biologically indolent nature of appendiceal NETs that are less than 5 mm [18] may also be explained by their increased proportion of L-cell tumors as identified in our series. Mixed tumors were intermediate in all of these parameters. Both EC-cell and mixed tumors had more frequent lymphatic and/or vascular invasion than L-cell tumors. The tumor type correlated with pT stage and the only patient with metastatic disease in this series had an EC-cell tumor. Unlike EC-cell tumors, L-cell tumors did not show metastatic nodal involvement. These data further support the findings of the only previous series that performed hormone stains [10]; that study also reported lack of nodal metastasis in patients with glucagon-positive appendiceal NETs (presumed to be L-cell tumors) compared to serotonin-positive tumors. Our findings indicate that tumor subtyping may be an important pathological variable for risk stratification and prognosis in patients with appendiceal NETs. Current guidelines do not include any reference to tumor cell type or hormone product in patient management [6, 19] but this should be reconsidered.

The importance of cell type in tumor prognosis is also associated with an important message for clinical surveillance. It is generally thought that patients with gastrointestinal NETs can undergo surveillance by measurement of either circulating serotonin or, more commonly, its metabolite urinary 5HIAA (5-hydroxyindoleacetic acid). While this may be true for patients with ileal NETs that are almost exclusively EC-cell tumors, our data show that this test would be misleading in almost half of patients with appendiceal NETs since their tumor did not produce abundant serotonin. Instead, they may have other circulating biomarkers including PYY, PP, and glucagon, which would be reliable only if proven to be diffusely and strongly expressed by the tumor.

Our data showing a less aggressive behavior of L-cell appendiceal NETs are similar to those obtained for rectal NETs where L-cell tumors have been shown to be less aggressive. Thus, a simple appendectomy would be sufficient for the treatment of virtually all appendiceal L-cell NETs. Moreover, biochemical surveillance using circulating L-cell biomarkers may be considered in patients with other questionable risk factors. Since L-cell NETs may rarely show high-risk features such as mesoappendix involvement and intermediate Ki67 proliferation index, it would be prudent to offer surveillance in such cases until more long-term follow-up data are available. Recent data have also identified a subset of rectal NETs that express somatostatin [20]; in this study, we did not examine other hormones but future studies should focus on this and other potential hormone products to further refine the prognosis and management of patients with appendiceal NETs.

In conclusion, our study confirms that appendiceal NETs are not a homogeneous tumor population and are composed of at least three types including EC-cell, L-cell, and mixed tumors, each with unique clinicopathologic characteristics. Cell subtype should be taken into consideration in risk stratification and patient management.

## Data Availability

The original data available on reasonable request.

## References

[1] Pape UF, Perren A, Niederle B (2012). ENETS Consensus Guidelines for the management of patients with neuroendocrine neoplasms from the jejuno-ileum and the appendix including goblet cell carcinomas. Neuroendocrinology.

[2] Pape UF, Niederle B, Costa F (2016). ENETS Consensus Guidelines for Neuroendocrine Neoplasms of the Appendix (Excluding Goblet Cell Carcinomas). Neuroendocrinology.

[3] Rault-Petit B, Do CC, Guyetant S (2019). Current Management and Predictive Factors of Lymph Node Metastasis of Appendix Neuroendocrine Tumors: A National Study from the French Group of Endocrine Tumors (GTE). Ann Surg.

[4] Ricci C, Ingaldi C, Alberici L (2019). Histopathological diagnosis of appendiceal neuroendocrine neoplasms: when to perform a right hemicolectomy?. A systematic review and meta-analysis. Endocrine.

[5] Brighi N, La RS, Rossi G (2020). Morphological Factors Related to Nodal Metastases in Neuroendocrine Tumors of the Appendix: A Multicentric Retrospective Study. Ann Surg.

[6] Mohamed A, Wu S, Hamid M et al. Management of Appendix Neuroendocrine Neoplasms: Insights on the Current Guidelines. Cancers (Basel) 2022; 15(1). 10.3390/cancers15010295 PMC9818268 36612291

[7] Bednarczuk T, Zemczak A, Bolanowski M (2022). Neuroendocrine neoplasms of the small intestine and the appendix - update of the diagnostic and therapeutic guidelines (recommended by the Polish Network of Neuroendocrine Tumours) [Nowotwory neuroendokrynne jelita cienkiego i wyrostka robaczkowego - uaktualnione zasady diagnostyki i leczenia (rekomendowane przez Polska Siec Guzow Neuroendokrynnych)]. Endokrynol Pol.

[8] Holmager P, Langer SW, Kjaer A (2024). Appendiceal Neuroendocrine Neoplasms: an Update for 2023. Curr Oncol Rep.

[9] Kuhlen M, Kunstreich M, Pape UF et al. Lymph node metastases are more frequent in paediatric appendiceal NET >/=1.5 cm but without impact on outcome - Data from the German MET studies. Eur J Surg Oncol 2024; 50(4):108051. 10.1016/j.ejso.2024.108051 38430702

[10] Holmager P, Willemoe GL, Nielsen K (2021). Neuroendocrine neoplasms of the appendix: Characterization of 335 patients referred to the Copenhagen NET Center of Excellence. Eur J Surg Oncol.

[11] Asa SL, Mete O, Cusimano MD (2021). Pituitary neuroendocrine tumors: a model for neuroendocrine tumor classification. Mod Pathol.

[12] Lee SH, Kim BC, Chang HJ (2013). Rectal neuroendocrine and L-cell tumors: diagnostic dilemma and therapeutic strategy. Am J Surg Pathol.

[13] Sohn JH, Cho MY, Park Y (2015). Prognostic Significance of Defining L-Cell Type on the Biologic Behavior of Rectal Neuroendocrine Tumors in Relation with Pathological Parameters. Cancer Res Treat.

[14] Kim JY, Kim KS, Kim KJ (2015). Non-L-cell immunophenotype and large tumor size in rectal neuroendocrine tumors are associated with aggressive clinical behavior and worse prognosis. Am J Surg Pathol.

[15] Matsukuma KE, Montgomery EA (2012). Tubular carcinoids of the appendix: the CK7/CK20 immunophenotype can be a diagnostic pitfall. J Clin Pathol.

[16] Kim J, Kim JY, Oh EH (2020). Chromogranin A Expression in Rectal Neuroendocrine Tumors Is Associated With More Aggressive Clinical Behavior and a Poorer Prognosis. Am J Surg Pathol.

[17] Bellizzi AM (2020). SATB2 in neuroendocrine neoplasms: strong expression is restricted to well-differentiated tumours of lower gastrointestinal tract origin and is most frequent in Merkel cell carcinoma among poorly differentiated carcinomas. Histopathology.

[18] Noor M, Huber AR, Cates JMM, Gonzalez RS (2021). Risk factors for progression of appendiceal neuroendocrine tumours: low-stage tumours <5 mm appear to be overwhelmingly indolent and may merit a separate designation. Histopathology.

[19] Nesti C, Brautigam K, Benavent M (2023). Hemicolectomy versus appendectomy for patients with appendiceal neuroendocrine tumours 1–2 cm in size: a retrospective, Europe-wide, pooled cohort study. Lancet Oncol.

[20] Kim J, Yang DH, Jung H (2023). Clinicopathologic Impact of Peptide Hormonal Expression in Rectal Neuroendocrine Tumors. Arch Pathol Lab Med.

